# Burden of Obesity Among Adults in the United States: A Population Health Study

**DOI:** 10.3390/healthcare14142078

**Published:** 2026-07-11

**Authors:** Monira Alwhaibi

**Affiliations:** Department of Clinical Pharmacy, College of Pharmacy, King Saud University, Riyadh 11149, Saudi Arabia; malwhaibi@ksu.edu.sa

**Keywords:** obesity, health-related quality of life, humanistic burden, chronic disease, health disparities, MEPS, public health, United States

## Abstract

**Background**. As a growing public health concern, obesity places a significant burden on both healthcare systems and society. While its association with chronic diseases is well documented, less is known about its broader impact on health-related quality of life (HRQoL) in the general population. This study investigated the clinical and humanistic burden of obesity among U.S. adults using nationally representative data. **Methods.** This cross-sectional study utilized data from the 2019–2022 Medical Expenditure Panel Survey (MEPS). Adults aged ≥18 years with complete BMI and HRQoL information were eligible for inclusion. Based on the World Health Organization BMI criteria, participants were categorized as underweight, normal weight, overweight, or obese. Associations between obesity, sociodemographic factors, chronic diseases, and HRQoL were examined using descriptive analyses, chi-square tests, one-way ANOVA, and multinomial logistic regression. **Results.** The study included 9311 adults, of whom 35.0% had obesity, 31.0% were overweight, 31.9% had normal weight, and 1.8% were underweight. Health-related quality of life differed significantly across BMI categories. Participants with obesity had the lowest mean Physical Component Summary (PCS) score (48.84 ± 0.23) compared with those with normal weight (54.28 ± 0.19; *p* < 0.0001), as well as lower mean Mental Component Summary (MCS) scores (49.77 ± 0.23 vs. 51.37 ± 0.23; *p* < 0.0001). After adjustment for potential confounders, higher PCS scores were associated with reduced odds of obesity (adjusted odds ratio [AOR] = 0.96, 95% confidence interval [CI]: 0.95–0.97). A higher odds of obesity was observed among those with diabetes (AOR = 3.43, 95% CI = 2.48–4.77), hypertension (AOR = 2.52, 95% CI = 2.07–3.06), osteoarthritis (AOR = 1.43, 95% CI = 1.01–2.02), depression (AOR = 1.45, 95% CI = 1.11–1.88), and anxiety (AOR = 1.40, 95% CI = 1.11–1.76). **Conclusions.** More than one-third of participants had obesity, which was associated with substantial clinical and humanistic burden. Individuals with obesity experienced significantly poorer physical-health-related quality of life and a greater burden of chronic physical and mental health conditions. These results underscore the need for integrated interventions aimed at preventing and managing obesity while improving health-related quality of life.

## 1. Introduction

Obesity is a major global epidemic and one of the leading contributors to morbidity and premature mortality worldwide [1,2,3,4,5]. The prevalence of obesity has increased substantially over recent years among adults across both developed and developing countries, creating a significant burden on healthcare systems and society. In 2022, more than one billion people worldwide were living with obesity, accounting for approximately 13% of the global population [2]. The prevalence of obesity has increased dramatically; the current projections suggest that by 2035, approximately 1.9 billion adults will have obesity, equivalent to one-quarter of the world’s population. By 2050, this number is expected to rise to 3.8 billion adults, representing more than half of the projected global adult population [2].

Obesity is associated with many chronic conditions, including diabetes, cardiovascular disease, dyslipidemia, osteoarthritis, respiratory disorders, cancer, and several mental health conditions [6]. These obesity-related comorbidities contribute to reduced life expectancy, impaired physical functioning, and increased healthcare costs and expenditures [7,8]. In addition to its clinical burden, obesity imposes a substantial economic burden on healthcare and society [9,10,11]. Obese individuals incurred medical costs that were about 30% higher than those of their normal-weight counterparts [11].

Beyond its clinical and economic burden, obesity also exerts a substantial humanistic burden through its negative effects on health-related quality of life (HRQoL) [9,12,13,14,15,16,17,18,19,20,21]. Individuals with obesity frequently experience limitations in mobility, reduced physical and social functioning, emotional distress, and poorer health status. Health-related quality of life is increasingly recognized as an important patient-centered outcome that reflects the broader impact of chronic conditions on physical, mental, and social well-being [22].

Obesity is strongly influenced by social and demographic determinants, with certain populations experiencing a disproportionate burden of disease and related health outcomes. For example, factors such as age, sex, race/ethnicity, socioeconomic status, education, employment, and access to healthcare may influence obesity prevalence and its associated clinical and humanistic burdens [23,24,25]. Globally, obesity disproportionately affects racial and ethnic minority populations and individuals with lower socioeconomic status, highlighting the importance of understanding the broader social determinants associated with obesity.

Despite extensive research on obesity, there remains a need for contemporary national-level evidence that simultaneously evaluates the clinical and humanistic burden of obesity and identifies populations that may experience disproportionate adverse outcomes. Racial/ethnic minorities and individuals with lower socioeconomic status often face a higher prevalence of obesity and obesity-related complications; however, disparities in the burden of obesity across these groups have not been fully characterized at the population level. In addition, while obesity is associated with numerous chronic physical and mental health conditions, the relative contribution of these comorbidities to impaired HRQoL remains insufficiently understood. Therefore, the objective of this study was to examine the clinical and humanistic burden of obesity among U.S. adults using nationally representative MEPS data. Specifically, we assessed the associations between obesity, HRQoL, and obesity-related comorbidities, and explored disparities by race/ethnicity and income to identify populations at greatest risk of obesity.

## 2. Methods

### 2.1. Study Population and Data

A cross-sectional study design was conducted using survey data from the Medical Expenditure Panel Survey (MEPS), a national survey of the United States (U.S.) population. Eligible adults were aged 18 years and above, had no missing data on body mass index (BMI), were alive over the course of the study duration, and had no missing data on quality of life. We utilized data across four years from 2019 to 2022. After applying the inclusion criteria, the final sample was 9311. Chronic conditions in the MEPS are identified based on self-reported medical conditions that are subsequently coded by professional coders using ICD-10-CM codes.

### 2.2. Measures

#### 2.2.1. Dependent Variable: Obesity Group

Based on body mass index (BMI), adults were categorized into four groups according to World Health Organization criteria as (1) underweight (<18.5 kg/m^2^), (2) normal weight (18.5–24.9 kg/m^2^), (3) overweight (25.0–29.9 kg/m^2^), and (4) obese (≥30.0 kg/m^2^) [26,27].

#### 2.2.2. Key Independent Variable (Health-Related Quality of Life)

Health-related Quality of Life (HRQoL) was evaluated in the survey using the Veterans RAND 12 Item Health Survey (VR-12©), a self-report tool that consists of eight health domains. These domains were split into two categories: (1) a Physical Component Summary (PCS), and (2) a Mental Component Summary (MCS) [28]. The PCS comprises the following items: physical functioning, physical activity, pain, and general health, while the MCS comprises vitality, social functioning, emotional functioning, and mental health. Responses to these items were measured on a five-point Likert scale ranging from “No, none of the time” to “Yes, all of the time”. The VR-12 is a reliable scale with a strong internal consistency of nearly 0.85 for the PCS-12 and 0.83 for the MCS-12 [29,30,31,32].

#### 2.2.3. Other Independent Variables

Independent variables included sociodemographic information (age, gender, race/ethnicity, marital status, region of residence, employment, educational level, and household income), perceived physical health, health and prescription insurance status, and physical activity. Additionally, we evaluated some common chronic conditions known to be related to obesity, including heart disease, hypertension, diabetes, hyperlipidemia, asthma, chronic obstructive pulmonary disease, gastroesophageal reflux disease, osteoarthritis, thyroid disorders, cancer, anxiety, and depression.

### 2.3. Statistical Analyses

Descriptive statistics were used to describe the study population. Prior to inferential analyses (i.e., chi-square tests, one-way analysis of variance (ANOVA)), the distribution of continuous variables was assessed for normality using graphical methods and appropriate normality tests. Because the HRQoL scores were approximately normally distributed and the study included a large nationally representative sample, parametric tests (one-way ANOVA) were used to compare mean scores across BMI groups. Categorical variables were compared using chi-square tests. To evaluate the association between health-related quality of life, logistic regression analysis was employed. This model adjusted for covariates, including sociodemographic variables, insurance, physical activity, self-perceived physical health, and coexisting chronic health conditions. Analyses were conducted using the survey procedures in SAS 9.4 (SAS Institute Inc., Cary, NC, USA).

## 3. Results

### 3.1. Characteristics of the Study Sample

The study sample included 9311 participants, of whom 1.8% were underweight, 31.9% normal weight, 31.0% overweight, and 35.0% obese (Table 1). Overall, obesity represented the largest weight category in the sample. Age was significantly associated with weight status: younger adults (18–29 years) had the highest proportion of normal weight (43.9%) and the lowest obesity prevalence (26.2%), whereas older age groups showed higher obesity rates, reaching 38.8% among those aged 50–59 years. Gender differences, race/ethnicity, and regional variations were also observed. Socioeconomic factors (income, educational attainment, employment status) were significantly associated with obesity. Access to healthcare (insurance) was associated with weight status. Health status and behaviors were strongly associated with obesity. Participants reporting fair/poor health had higher obesity prevalence compared to those reporting excellent/very good health. Physical inactivity was also linked to obesity, with 40.1% obesity among those reporting no exercise versus 27.7% among those exercising ≥3 times/week. Chronic conditions were consistently more prevalent among obese individuals. Obesity prevalence was notably higher among those with heart disease, diabetes, hypertension, hyperlipidemia, osteoarthritis, GERD, asthma, COPD, depression and anxiety.

### 3.2. Mean Health-Related Quality of Life by Obesity Group

Weighted mean scores for health-related quality of life, including the Physical Component Summary (PCS) and Mental Component Summary (MCS), differed significantly across obesity groups (*p* < 0.0001 for both) (Table 2).

Physical Health (PCS): The overall mean PCS score for the sample was 51.01 (SD = 9.67). Significant differences were observed across weight categories. Individuals with normal weight had the highest PCS scores (54.28 ± 0.19), indicating better physical health status. This was followed by those who were overweight (52.47 ± 0.21) and underweight (51.68 ± 0.94), respectively. In contrast, individuals with obesity had the lowest PCS scores (48.84 ± 0.23), suggesting substantially poorer physical-health-related quality of life compared to all other groups.

Mental Health (MCS): The overall mean MCS score was 50.67 (SD = 9.30). Differences across obesity groups were also statistically significant. Participants who were overweight (51.49 ± 0.21) and normal weight (51.37 ± 0.23) reported the highest mental health scores. Lower MCS scores were observed among individuals who were obese (49.77 ± 0.23) and underweight (49.28 ± 0.85), indicating comparatively poorer mental-health-related quality of life in these groups.

### 3.3. Adjusted Association Between HRQoL and Obesity Groups

Multinomial logistic regression analysis was conducted to examine factors associated with underweight, overweight, and obesity, using normal weight as the reference group. Adjusted odds ratios (AORs) with 95% confidence intervals (CIs) are presented in Table 3.

#### 3.3.1. Health-Related Quality of Life (HRQoL)

Higher physical health (PCS) scores were significantly associated with lower odds of being overweight (AOR = 0.98; 95% CI: 0.97–1.00) and obese (AOR = 0.96; 95% CI: 0.95–0.97), indicating that better physical health reduces the likelihood of non-normal weight status. In contrast, mental health (MCS) was not significantly associated with weight status across groups.

#### 3.3.2. Sociodemographic Factors

Age showed a differential association with weight status. Compared to adults aged 60–64 years, younger adults (18–29 years) had lower odds of being overweight (AOR = 0.67; 95% CI: 0.52–0.86), while adults aged 30–59 years had higher odds of obesity, particularly those aged 40–49 years (AOR = 1.67; 95% CI: 1.33–2.10).

Gender differences were observed, with women more likely to be overweight (AOR = 1.88; 95% CI: 1.65–2.14) and obese (AOR = 1.23; 95% CI: 1.07–1.42) compared to men. Significant racial/ethnic disparities were identified. African American individuals had higher odds of being overweight (AOR = 1.71) and obese (AOR = 1.76), and Latinos/Latinas also had increased odds of being overweight (AOR = 1.43) and obese (AOR = 1.23) compared to Whites. Marital status was associated with increased risk of excess weight, as married individuals had higher odds of being overweight (AOR = 1.37) and obese (AOR = 1.35) compared to individuals who had never married. Individuals with a high income had reduced odds of obesity (AOR = 0.73; 95% CI: 0.55–0.98) compared to those who were poor. Employment status was significantly associated with weight status. Employed individuals had higher odds of being overweight (AOR = 1.42) and obese (AOR = 1.66) compared to those who were not employed.

#### 3.3.3. Health Status and Behaviors

Individuals reporting excellent/very good health had significantly lower odds of being overweight (AOR = 0.73) and obese (AOR = 0.53) compared to those reporting fair/poor health. Physical activity was a protective factor against excess weight. Participants engaging in physical activity ≥3 times per week had lower odds of being overweight (AOR = 0.86) or obese (AOR = 0.68) compared to those reporting no exercise.

#### 3.3.4. Clinical Characteristics

Several chronic conditions were significantly associated with obesity. Individuals with hypertension were twofold more likely to be obese (AOR = 2.52; 95% CI: 2.07–3.06), and those with diabetes had over threefold higher odds of obesity (AOR = 3.43; 95% CI: 2.48–4.77). Diabetes was also associated with increased odds of being overweight (AOR = 1.72). Osteoarthritis was associated with higher odds of obesity (AOR = 1.43), while depression (AOR = 1.45) and anxiety (AOR = 1.40) were also significantly associated with higher odds of obesity.

## 4. Discussion

This nationally representative study examined the clinical and humanistic burden of obesity among U.S. adults. Several important findings emerged. First, obesity was highly prevalent, affecting more than one-third of adults in our sample. Also, obesity was associated with a substantially higher prevalence of multiple chronic conditions, including hypertension, diabetes, osteoarthritis, depression, and anxiety. In addition, individuals with obesity reported significantly poorer physical- and mental-health-related quality of life than those with normal weight.

The prevalence of obesity observed in this study is consistent with national estimates in the United States [33]. The finding that obesity was more common among women, African American adults, lower-income individuals, and residents of the South is also consistent with previous research documenting significant sociodemographic disparities in obesity prevalence [34,35,36]. These disparities can be attributed to access to healthy foods, healthcare access, neighborhood environments, and socioeconomic resources.

Our findings show the substantial clinical burden associated with obesity. Obese individuals were more likely to have hypertension, diabetes, osteoarthritis, and mental health conditions, even after adjustment for other covariates, with obese adults demonstrating more than threefold higher odds of diabetes and more than twofold higher odds of hypertension. These findings are consistent with the well-established pathophysiological relationship between excess adiposity, insulin resistance, metabolic disorders, and cardiovascular risk [37]. The strong association between obesity and osteoarthritis may be explained by both mechanical stress on weight-bearing joints and obesity-related inflammatory processes.

Although the present study used nationally representative U.S. data, the findings have important implications for Saudi Arabia, where obesity has reached epidemic proportions. National studies from Saudi Arabia have similarly reported high obesity prevalence and strong associations with diabetes, hypertension, cardiovascular disease, and impaired quality of life [38]. While differences in healthcare systems, demographics, and lifestyle factors should be considered when comparing populations, the consistency of these findings suggests that the clinical and humanistic burden of obesity is a global public health challenge. Future studies using nationally representative Saudi data are warranted to directly quantify these relationships within the Saudi population.

An important contribution of this study is the evaluation of obesity’s humanistic burden through HRQoL measures. Individuals with obesity had significantly lower PCS and MCS scores compared with normal-weight adults, indicating poorer physical and mental well-being. However, after adjustment for sociodemographic characteristics, comorbid conditions, and other covariates, only the physical component score remained significantly associated with obesity. This finding suggests that the negative impact of obesity on quality of life may be driven primarily through physical and functional limitations, pain, and reduced mobility rather than through mental health status. Similar findings have been reported in a previous study demonstrating a stronger relationship between obesity and physical domains of HRQoL, but not with the mental domain [14].

The observed association between obesity and depression and anxiety further highlights the complex relationship between physical and psychological health. Although MCS scores of HRQoL were not associated with obesity in adjusted analyses, obese individuals were more likely to suffer from depression and anxiety. The relationship could be bidirectional, whereby obesity increases psychological distress through stigma and functional limitations, while mental health conditions may contribute to unhealthy behaviors and weight gain. In addition, physical activity emerged as an important protective factor. Adults who engaged in physical activity at least three times per week had significantly lower odds of obesity compared with those reporting no exercise. This finding supports existing evidence demonstrating the importance of regular physical activity in obesity prevention and management. Interventions that promote physical activity may therefore improve weight reduction, which has been shown to improve quality of life [39].

Our findings indicating a lower prevalence of obesity among individuals with higher income are consistent with previous studies. This relationship may be explained by socioeconomic factors that shape lifestyle behaviors and access to healthcare. Individuals with lower socioeconomic status often experience reduced access to healthy foods, fewer opportunities for physical activity, greater food insecurity, limited healthcare access, and environmental barriers that contribute to obesity. Conversely, individuals with higher income generally have greater access to healthier dietary choices, preventive healthcare services, recreational facilities, and health education, which may contribute to a lower prevalence of obesity. These socioeconomic inequalities may also partially explain the racial and ethnic disparities observed in our study. Racial and ethnic differences in obesity are likely multifactorial and reflect the complex interplay of biological, behavioral, socioeconomic, and environmental factors. Individuals from racial and ethnic minority groups may be disproportionately affected by social determinants of health, including lower socioeconomic status, reduced access to healthy foods, limited access to preventive healthcare, and neighborhood environments that do not support healthy lifestyles. Cultural dietary practices, chronic psychosocial stress, experiences of structural inequities, and differences in health literacy may also contribute to disparities in obesity prevalence. Although genetic susceptibility may influence individual risk, current evidence suggests that social and environmental determinants play a greater role in explaining differences in obesity [40]. These findings underscore the importance of developing culturally appropriate and equitable obesity prevention and management strategies that address both individual behaviors and the broader social determinants of health.

### 4.1. Implications

#### 4.1.1. Clinical and Research Implications

The findings emphasize the importance of routine obesity screening in clinical practice. Healthcare providers should recognize obesity as a chronic disease requiring long-term management rather than solely focusing on weight reduction. Given the strong associations with diabetes, hypertension, osteoarthritis, depression, and anxiety, multidisciplinary approaches integrating medical, behavioral, and lifestyle interventions may be necessary to address the broad burden of obesity. Incorporating HRQoL assessments into routine care may also help identify patients experiencing substantial functional limitations and support more patient-centered treatment decisions.

Future studies should employ longitudinal designs to better understand causal relationships between obesity, chronic diseases, and quality of life outcomes. Research examining obesity severity, duration of obesity, and weight change over time may provide additional insight into factors influencing HRQoL.

#### 4.1.2. Public and Policy Implications

The high prevalence of obesity and its association with poorer quality of life underscore the need for population-level prevention strategies. Public health programs should focus on promoting healthy dietary behaviors, increasing opportunities for physical activity, and reducing barriers to healthy lifestyles. Targeted interventions may be particularly beneficial for high-risk populations identified in this study, including racial and ethnic minority groups and individuals with a lower socioeconomic status.

The findings also support policies aimed at addressing social determinants of obesity and improving access to preventive healthcare services. Given the substantial burden of obesity-related chronic conditions, investments in prevention may reduce long-term healthcare expenditures and improve population health outcomes.

### 4.2. Study Limitations

Several limitations should be considered when interpreting these findings. First, the cross-sectional design of the MEPS precludes causal inference. Associations observed between obesity, comorbidities, and HRQoL cannot establish temporal relationships or directionality. Second, BMI was based on self-reported height and weight, which may be subject to recall bias. Third, BMI does not distinguish between fat mass and lean body mass and may not fully capture body composition or central adiposity. Moreover, certain clinical conditions affecting BMI such as pregnancy, lactation, or menopausal status could not be accounted for because of the limitations of the secondary dataset. Also, laboratory biomarkers such as HbA1c, lipid profile, and triglyceride levels would provide additional clinical insight; however, the MEPS database does not collect these laboratory measurements. Fourth, although multiple confounders were controlled for, residual confounding from unmeasured variables such as dietary patterns, environmental factors, or genetic predisposition may remain.

Despite these limitations, the study has several strengths, including the use of a large nationally representative sample, validated measures of health-related quality of life, and comprehensive adjustment for demographic, socioeconomic, behavioral, and clinical factors. These strengths provide robust evidence regarding the substantial clinical and humanistic burden associated with obesity among U.S. adults.

## 5. Conclusions

Obesity remains a highly prevalent condition among U.S. adults and is associated with a substantial clinical and humanistic burden. In this nationally representative study, individuals with obesity experienced significantly poorer health-related quality of life and had a higher prevalence of several chronic conditions, including diabetes, hypertension, osteoarthritis, depression, and anxiety. Sociodemographic disparities in obesity prevalence were also evident, highlighting the influence of broader social determinants of health. These findings underscore the importance of recognizing obesity as a chronic disease that extends beyond excess body weight and affects multiple dimensions of health and well-being. Comprehensive prevention and management strategies that incorporate lifestyle modification, chronic disease management, mental health support, and quality of life assessment are needed to reduce the burden of obesity and improve health outcomes among adults.

## Figures and Tables

**Table 1:** Characteristics of the Study Sample (n = 9311); Number and Raw Percentage by Weight Group.

		Total		Underweight		Normal		Overweight		Obese			
		N	Wt.%	N	Wt.%	N	Wt.%	N	Wt.%	N	Wt.%	χ^2^	*p*-Value
**All**		9311	100.0	168	1.8	2828	31.9	2909	31.0	3406	35.0		
**Age in years**													
	18–29	1679	21.6	66	3.7	723	43.9	433	26	457	26.2	194.5	<0.0001
	30–39	1958	21.4	37	1.9	647	33.7	565	29	709	35.2		
	40–49	1939	20.3	19	1.0	510	27.6	641	33	769	38.1		
	50–59	2357	23.4	22	0.9	592	26.3	804	34	939	38.8		
	60–64	1378	13.2	24	1.5	356	26.2	466	34	532	38.0		
**Gender**													
	Women	5071	52.1	173	2.3	2445	34.9	2080	27	2833	35.4	163.7	<0.0001
	Men	4240	47.9	96	1.5	1686	27.8	2423	39	1986	32.0		
**Race/ethnicity**													
	White	5133	62.3	151	1.8	2628	32.3	2752	32	2855	33.7	210.7	<0.0001
	African American	1227	11.5	29	1.6	370	20.7	528	33	804	44.6		
	Latino/Latina	2094	16.5	47	1.6	640	27.2	902	37	922	34.7		
	Others	857	9.7	42	4.0	493	46.1	321	30	238	20.1		
**Marital status**													
	Married	4637	53.0	98	1.4	1927	29.1	2489	36	2354	33.6	100.7	<0.0001
	Wid./Div./Sep.	1732	14.9	75	2.2	1061	30.6	1145	31	1328	35.9		
	Never married	2942	32.1	96	3.0	1143	37.7	869	27	1137	32.4		
**Region**													
	Northeast	1359	16.3	43	2.2	680	34.0	689	33	692	31.2	31.7	<0.0001
	Midwest	2005	22.9	52	1.8	880	29.7	965	33	1100	35.9		
	South	3394	36.1	96	1.8	1386	29.4	1605	33	1902	36.2		
	West	2553	24.7	78	2.3	1185	35.0	1244	33	1125	29.9		
**Education Level**													
	LT HS	336	2.4	18	3.3	131	25.7	215	38	208	33.0	31.2	<0.0001
	HS	915	7.8	33	2.8	353	30.9	363	30	486	36.2		
	>HS	8013	89.4	214	1.8	3633	31.9	3894	33	4086	33.5		
**Employment**													
	Employed	6865	77.9	120	1.5	2394	31.6	2596	33	2702	34.2	18.3	<0.0001
	Not employed	2441	22.1	149	2.7	1734	31.6	1906	33	2115	33.2		
**Poverty status**													
	Poor	1270	9.7	53	3.3	494	28.0	538	30	735	39.1	86.1	<0.0001
	Near Poor	1583	13.3	44	1.7	708	29.2	778	31	987	38.0		
	Middle Income	2445	26.0	72	1.9	992	27.9	1277	34	1333	36.2		
	High Income	4013	51.1	100	1.8	1937	35.0	1910	33	1764	30.3		
**Rx Insurance**													
	Rx insurance	5636	67.3	106	1.6	2186	33.3	2231	32	2302	33.1	22.9	<0.0001
	No Rx insurance	3675	32.7	163	2.4	1945	29.3	2272	34	2517	34.8		
**Health Insurance**													
	Private	6450	75.6	132	1.6	2661	32.8	2800	33	2881	33.1	28.8	<0.0001
	Public	1937	16.2	117	2.8	1188	28.5	1384	33	1620	36.1		
	Uninsured	924	8.1	20	2.0	282	31.0	319	35	318	32.5		
**General health**													
	Excellent/very good	5329	61.1	151	2.0	2738	38.1	2617	34	1993	25.9	472.6	<0.0001
	Good	2786	28.0	68	1.7	999	23.6	1364	32	1835	42.5		
	Fair/poor	1196	10.9	50	2.5	394	19.6	522	27	991	50.6		
**Physical activity**													
	3/week	4622	51.3	133	1.9	2371	36.3	2355	34	1945	27.7	201.1	<0.0001
	No exercise	4649	48.2	135	2.0	1747	26.7	2129	31	2852	40.1		
**Heart disease**													
	Yes	354	3.4	20	1.3	304	23.0	420	32	554	43.3	55.1	<0.0001
	No	8957	96.6	249	2.0	3827	32.4	4083	33	4265	32.9		
**Hypertension**													
	Yes	1759	17.1	61	1.4	832	20.1	1380	33	1976	45.9	323.5	<0.0001
	No	7552	82.9	208	2.2	3299	36.0	3123	33	2843	29.2		
**Diabetes**													
	Yes	836	7.8	14	0.9	236	13.3	525	30	1038	56.2	350.8	<0.0001
	No	8475	92.2	255	2.1	3895	33.9	3978	33	3781	30.9		
**Hyperlipidemia**													
	Yes	1257	12.4	36	1.0	686	22.3	1151	35	1411	41.8	137.5	<0.0001
	No	8054	87.6	233	2.2	3445	34.1	3352	32	3408	31.6		
**Asthma**													
	Yes	627	6.3	24	2.2	205	24.4	266	28	492	45.6	47.8	<0.0001
	No	8684	93.7	245	1.9	3926	32.1	4237	33	4327	33.0		
**COPD**													
	Yes	204	2.0	14	2.8	104	24.7	143	31	196	41.9	12.4	0.006
	No	9107	98.0	255	1.9	4027	31.8	4360	33	4623	33.6		
**Osteoarthritis**													
	Yes	489	4.6	25	2.2	262	21.2	358	30	623	47.2	82.5	<0.0001
	No	8822	95.4	244	1.9	3869	32.5	4145	33	4196	32.6		
**GERD**													
	Yes	490	5.0	17	1.6	224	21.2	366	33	536	44.3	55.1	<0.0001
	No	8821	95.0	252	2.0	3907	32.5	4137	33	4283	32.9		
**Cancer**													
	Yes	323	3.5	22	2.1	342	31.9	409	37	351	29.5	8.1	0.044
	No	8988	96.5	247	2.0	3789	31.6	4094	32	4468	34.2		
**Depression**													
	Yes	851	9.1	21	1.6	297	26.1	342	26	602	45.8	72.3	<0.0001
	No	8460	90.9	248	2.0	3834	32.1	4161	33	4217	32.6		
**Anxiety**													
	Yes	973	10.9	36	2.2	397	31.6	368	26	592	40.2	25.7	<0.0001
	No	8338	89.1	233	1.9	3734	31.6	4135	33	4227	33.0		
**Thyroid**													
	Yes	649	6.9	22	1.2	354	27.0	457	31	583	40.3	24.0	<0.0001
	No	8662	93.1	247	2.0	3777	32.1	4046	33	4236	33.1		

*p*-value represents significant differences in characteristics between obesity groups from chi-square tests. COPD: chronic obstructive pulmonary disease; GERD: gastroesophageal reflux disease; LT HS: less than high school; Wt: weighted; Rx: medication; Wid./Div./Sep.: widowed, divorced, or separated.

**Table 2:** Weighted means and standard errors from health-related quality of life scores by weight group among adults.

		Total Sample		Underweight		Normal		Overweight		Obese		
HRQoL		Mean	SD	Mean	SE	Mean	SE	Mean	SE	Mean	SE	*p*-Value
	**PCS**	51.01	9.67	51.68	0.94	54.28	0.19	52.47	0.21	48.84	0.23	<0.0001
	**MCS**	50.67	9.3	49.28	0.85	51.37	0.23	51.49	0.21	49.77	0.23	<0.0001

*p*-value represents significant mean differences by weight group using one-way ANOVA. HRQoL: health-related quality of life; MCS: mental component summary; PCS: physical component summary; SE: standard error; SD: standard deviation.

**Table 3:** Adjusted Odds Ratios and 95% Confidence Intervals from Multinomial Regression Analysis.

		Underweight		Overweight				Obese		
		AOR	95% CI	Sig.	AOR	95% CI	Sig.	AOR	95% CI	Sig.
**HRQoL**										
	PCS	0.97	[0.94, 1.00]	*	0.98	[0.97, 1.00]	**	0.96	[0.95, 0.97]	***
	MCS	0.98	[0.96, 1.00]		1.00	[0.99, 1.01]		0.99	[0.99, 1.00]	
**Age in years**										
	18–29	1.72	[0.84, 3.53]	*	0.67	[0.52, 0.86]	***	1.06	[0.80, 1.39]	*
	30–39	1.24	[0.58, 2.63]		0.81	[0.64, 1.01]		1.50	[1.21, 1.87]	**
	40–49	0.80	[0.40, 1.61]		1.06	[0.84, 1.32]	*	1.67	[1.33, 2.10]	***
	50–59	0.68	[0.34, 1.37]		1.05	[0.84, 1.31]	*	1.30	[1.04, 1.63]	
	60–64 (Ref.)									
**Gender**										
	Women	0.96	[0.65, 1.42]		1.88	[1.65, 2.14]	***	1.23	[1.07, 1.42]	**
	Men (Ref.)									
**Race/ethnicity**										
	African American	1.17	[0.55, 2.47]		1.71	[1.31, 2.23]	***	1.76	[1.36, 2.28]	***
	Latino/Latina	0.91	[0.51, 1.64]		1.43	[1.18, 1.74]	***	1.23	[1.03, 1.48]	***
	Others	1.64	[1.03, 2.61]	*	0.68	[0.55, 0.86]	***	0.38	[0.29, 0.50]	***
	White (Ref.)									
**Marital status**										
	Married	1.10	[0.52, 2.35]		1.37	[1.10, 1.72]	***	1.35	[1.10, 1.65]	***
	Wid./Div./Sep.	1.32	[0.58, 2.99]		0.93	[0.73, 1.19]	*	1.08	[0.85, 1.37]	
	Never married (Ref.)									
**Region**										
	Midwest	0.96	[0.52, 1.78]		1.22	[1.00, 1.48]		1.18	[0.92, 1.52]	
	Northeast	0.89	[0.44, 1.79]		1.03	[0.82, 1.30]		0.97	[0.76, 1.25]	
	South	0.79	[0.48, 1.30]		1.11	[0.90, 1.36]		1.15	[0.90, 1.47]	
	West (Ref.)									
**Education Level**										
	LT HS	1.78	[0.63, 4.99]		1.21	[0.78, 1.89]		0.79	[0.53, 1.16]	**
	HS	1.26	[0.72, 2.19]		0.97	[0.74, 1.25]	*	0.91	[0.72, 1.15]	**
	>HS (Ref.)									
**Employment**										
	Employed	0.84	[0.53, 1.35]		1.42	[1.20, 1.69]	***	1.66	[1.38, 1.99]	***
	Not employed (Ref.)									
**Poverty status**										
	High Income	0.87	[0.33, 2.29]		0.81	[0.61, 1.07]	*	0.73	[0.55, 0.98]	***
	Middle Income	0.83	[0.35, 1.96]		1.09	[0.84, 1.40]	*	1.08	[0.81, 1.45]	
	Near Poor	0.70	[0.31, 1.58]		0.85	[0.66, 1.09]		0.98	[0.74, 1.30]	
	Poor (Ref.)									
**Rx Insurance**										
	Rx insurance	1.17	[0.58, 2.36]		1.16	[0.94, 1.43]		1.00	[0.80, 1.25]	
	No Rx insurance (Ref.)									
**Health Insurance**										
	Private	1.05	[0.39, 2.85]		1.07	[0.80, 1.42]		1.07	[0.79, 1.43]	
	Public	1.42	[0.66, 3.03]		1.10	[0.84, 1.43]		1.18	[0.90, 1.56]	
	Uninsured (Ref.)									
**General health**										
	Excellent/very good	1.08	[0.64, 1.80]		0.73	[0.62, 0.86]	*	0.53	[0.45, 0.63]	***
	Good	1.23	[0.64, 2.39]		0.86	[0.64, 1.18]		0.80	[0.60, 1.06]	
	Fair/poor (Ref.)									
**Physical activity**										
	3/week	0.90	[0.62, 1.30]		0.86	[0.74, 0.98]		0.68	[0.60, 0.78]	**
	No exercise									
**Heart disease**										
	Yes	0.15	[0.03, 0.70]	*	0.93	[0.61, 1.42]		1.23	[0.80, 1.88]	
**Hypertension**										
	Yes	1.54	[0.79, 3.03]		1.40	[1.13, 1.75]	**	2.52	[2.07, 3.06]	***
**Diabetes**										
	Yes	0.94	[0.33, 2.71]		1.72	[1.17, 2.54]	**	3.43	[2.48, 4.77]	***
**Hyperlipidemia**										
	Yes	1.39	[0.51, 3.74]		0.86	[0.66, 1.11]		0.94	[0.73, 1.20]	
**Asthma**										
	Yes	1.39	[0.79, 2.45]		1.04	[0.75, 1.45]		1.12	[0.82, 1.52]	
**COPD**										
	Yes	1.49	[0.56, 3.98]		1.00	[0.57, 1.74]		0.65	[0.37, 1.15]	
**Osteoarthritis**										
	Yes	1.63	[0.74, 3.58]		0.93	[0.63, 1.37]		1.43	[1.01, 2.02]	*
**GERD**										
	Yes	1.45	[0.68, 3.09]		1.09	[0.76, 1.57]		1.15	[0.81, 1.62]	
**Cancer**										
	Yes	1.23	[0.51, 3.00]		1.05	[0.76, 1.44]		0.74	[0.52, 1.06]	
**Depression**										
	Yes	0.76	[0.34, 1.70]		1.18	[0.91, 1.54]		1.45	[1.11, 1.88]	**
**Anxiety**										
	Yes	1.44	[0.74, 2.80]		1.39	[1.06, 1.84]	*	1.40	[1.11, 1.76]	**
**Thyroid**										
	Yes	1.69	[0.65, 4.39]		0.85	[0.63, 1.16]		0.78	[0.57, 1.06]	

Asterisks denote statistical significance from multinomial regression analysis. Reference group for Obesity = Normal Weight. *** *p* < 0.001; ** 0.001 ≤ *p* < 0.01; * 0.01 ≤ *p* < 0.05. AOR: adjusted odds ratio; CI: confidence interval; LT: less than; GT: greater than; LT HS: less than high school; Rx: medication; Ref: reference group; Sig: significance. Wid./Div./Sep.: widowed, divorced, or separated.

## Data Availability

The data utilized in this research are publicly accessible for researchers from the MEPS website: https://meps.ahrq.gov.
